# A Hydrogel-Based Electronic Skin for Touch Detection Using Electrical Impedance Tomography

**DOI:** 10.3390/s23031571

**Published:** 2023-02-01

**Authors:** Huiyang Zhang, Anubha Kalra, Andrew Lowe, Yang Yu, Gautam Anand

**Affiliations:** Institute of Biomedical Technologies, Auckland University of Technology, Auckland 1010, New Zealand

**Keywords:** touch sensor, electronic skin, impedance tomography, hydrogel sensor, human-machine interface, soft sensor, soft interface, tomographic imaging, soft robotics

## Abstract

Recent advancement in wearable and robot-assisted healthcare technology gives rise to the demand for smart interfaces that allow more efficient human-machine interaction. In this paper, a hydrogel-based soft sensor for subtle touch detection is proposed. Adopting the working principle of a biomedical imaging technology known as electrical impedance tomography (EIT), the sensor produces images that display the electrical conductivity distribution of its sensitive region to enable touch detection. The sensor was made from a natural gelatin hydrogel whose electrical conductivity is considerably less than that of human skin. The low conductivity of the sensor enabled a touch-detection mechanism based on a novel short-circuiting approach, which resulted in the reconstructed images being predominantly affected by the electrical contact between the sensor and fingertips, rather than the conventionally used piezoresistive response of the sensing material. The experimental results indicated that the proposed sensor was promising for detecting subtle contacts without the necessity of exerting a noticeable force on the sensor.

## 1. Introduction

An electronic skin (E-skin) is a conformable transducer that imitates the sensory function of the human skin. E-skins are present in various forms for detecting a diverse range of stimuli, including touch, compression, stretching, bending, and temperature [1]. In particular, enabling touch sensing could benefit applications such as soft interfaces for wearable health monitoring devices [2], sensory perception for smart prosthetics [3], and robots for assisting aged caregiving [4]. In the past decade, researchers have reported touch sensors based on various means, such as piezoresistive or capacitive sensor arrays that measure electrical resistance or capacitance distribution [2], optical sensors that measure reflected infrared light [5], and acoustic wave sensors that measure mechanical vibration [6]. Recently, E-skins based on electrical impedance tomography (EIT) have attracted considerable research interest due to their cost-effectiveness.

EIT was originally invented as a non-invasive biomedical imaging technique for producing cross-sectional images that illustrate the electrical conductivity distribution of the human body using current-voltage data measured from their boundary. Nowadays, the technique has been integrated with piezoresistive materials [7] to realize E-skins that can localize touch positions. When a touch is applied to such sensors, the electrical conductivity of the piezoresistive material changes in correspondence to its mechanical deformation caused by the exerted pressure. This change in electrical conductivity results in a perturbation of the current-voltage data. The perturbation provides information for the reconstruction algorithms of EIT to calculate the change in the conductivity distribution. Finally, the touch points can be localized based on the obtained conductivity images. Past works typically utilized conductive nanocomposites or fabrics as their sensing material. For example, Tallman et al. employed carbon nanofiber (CNF) and polyurethane (PU) nanocomposites for tactile imaging [8]. Yao et al. applied EIT to a piece of microfiber nonwoven fabrics [9]. Lee et al. proposed an E-skin made of multiwall carbon nanotubes (MWCNT) and silicone [10]. Some works utilized other types of materials. For instance, Chossat et al. developed a tactile sensor using a type of room-temperature liquid metal and ionic liquid [11]. Duan et al. proposed an E-skin made of conductive fabrics [12]. Due to the piezoresistive sensing mechanism, these sensors require compression with an adequate force to detect a touch. They may lack efficiency in detecting contacts applied using subtle or negligible forces.

In this paper, a new EIT-based electronic-skin sensor for touch detection using a short-circuiting approach is presented. The proposed sensor was fabricated using a flexible, non-toxic, and low-cost hydrogel. It could roughly localize contact positions and distinguish contact areas. The proposed method achieves EIT-based touch detection using the mismatch between the electrical conductivity of sensing materials and the contacting objects, which does not necessarily require the contacting objects to exert a force on the sensor. Compared to sensors proposed in previous works, the sensor reported in this work possesses the advantage of detecting subtle contacts made with a negligible force.

## 2. Methods and Materials

### 2.1. Electrical Impedance Tomography

**Figure 1:**
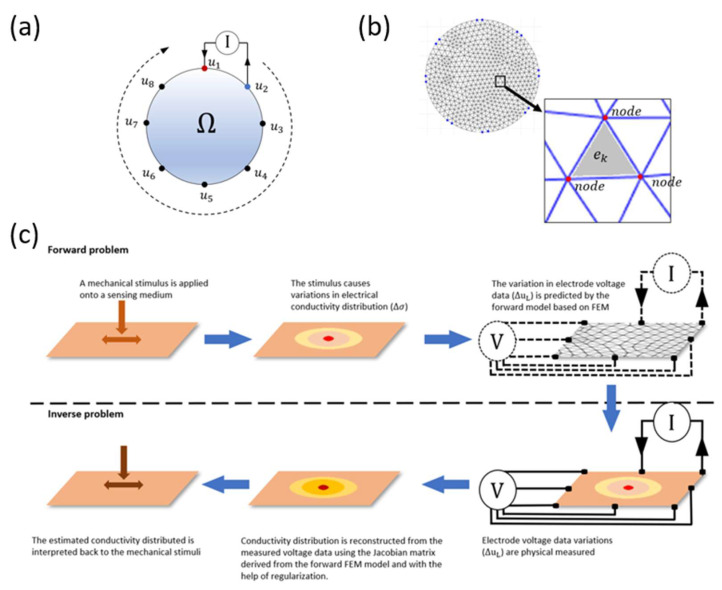
The working mechanism of EIT-based touch sensors. (**a**) The basic working principle of EIT: voltage measurements are taken at every electrode for each current injection. (**b**) Discretizing the domain of the sensing material into a collection of a finite number of elements and nodes using the finite element method. (**c**) The forward problem and the inverse problem.

The operating principle of the EIT technique adopted by E-skins is illustrated in Figure 1a. An electrically conductive film is attached with eight electrodes around its boundary. The interior of the film is denoted as Ω, and its boundary is donated as δΩ. An electric current is injected into the film via a selected electrode and sunk via another. The injected current produces an electric field that generates an electric potential distribution over the film. Meanwhile, the voltage at each electrode is measured with respect to a reference electrode. Then the current injection is switched to the next pair of electrodes. The electrode pairing typically follows either an adjacent or an opposite pattern [13]. Then voltage measurements are taken again from the new potential distribution. This procedure is repeated several times until the selected current injection pattern is completed. The voltage data recorded throughout the process is taken as the input to a reconstruction algorithm that estimates the conductivity distribution within the Ω.

The reconstruction algorithm consists of the forward problem and the inverse problem. The objective of the forward problem is to acquire a mathematical model for predicting the unknown electric potential distribution using a known current injection pattern and a known electrical conductivity distribution. The forward model will then serve as the foundation for the inverse problem, which allows the unknown conductivity distribution to be inversely calculated using measured voltage data. Derived from Maxwell’s equations, the governing equation of the forward problem is expressed in the form of an elliptical partial differential equation (PDE) [14], expressed as

(1) ∇·(−σ∇U)=0 in Ω

with a boundary condition (BC) described by

(2) $$\hat{n}\cdot \left(\sigma \nabla U\right)=g\;\mathrm{on}\;\delta \Omega \;$$

where σ denotes the point-wise conductivity distribution of the medium, g is the total amount of current that enters and leaves a point, and $\hat{n}$ is a unit normal vector pointing towards the exterior of the boundary. The physical meaning of the PDE and its BC is that the net current flowing into and out of any unique point within Ω is zero, and the net current can only be non-zero at points on δΩ where electrodes are attached. The problem is solved numerically using the finite element method (FEM), which transforms the PDE from its continuous form into a discrete form constructed with a collection of M elements connected by N nodes, as illustrated in Figure 1b. For each element, the PDE and the BC become a combined and discretized form [15], expressed as

(3) $$\overset{n}{\underset{i=1}{\sum }}\overset{n}{\underset{j=1}{\sum }}\left(\int_{\Omega }\sigma_{e}\left(\nabla \Phi_{i}\right)\cdot \left(\nabla \Phi_{j}\right)\;\mathrm{dA}\right)u_{i}=\overset{n}{\underset{i=1}{\sum }}\overset{n}{\underset{j=1}{\sum }}\left(\int_{\delta \Omega }{g\Phi }_{j}\mathrm{dS}\right)$$

where $\sigma_{e}$ denotes the element-wise conductivity, $\Phi_{i}$ and $\Phi_{j}$ are basis functions used in the FEM, i and j are node indices for the element, A is the area of the element, and S is the length of the boundary edge where it is applicable. Imposing a suitable electrode model and combining the amended local equations for all the elements results in a system of linear equations, which is expressed in its simplified form [14,15] as

(4) $$\left[\begin{array}{cc} A_{a}+A_{\beta } & A_{\gamma }\\ A_{\gamma }^{T} & A_{\delta } \end{array}\right]\left[\begin{array}{c} u_{n}\\ u_{l} \end{array}\right]=\left[\begin{array}{c} 0\\ I \end{array}\right]$$

where $u_{n}$ represents nodal voltage data, $u_{l}$ contains the voltage at each electrode, I is the current injection pattern, and the A matrices contain the information on element conductivity, the finite element model described by the left side of (3), and the contact impedance of the electrodes. Using (4), the electrode voltages $u_{l}$, as the solution to the forward problem, can thus be determined by solving the system of linear equations with a known current injection pattern I.

The inverse problem follows the opposite course by taking the measured electrode voltage data, $u_{l}$, as its input and inversely calculating the element-wise conductivity data contained in the A matrices. This is typically achieved by deriving a Jacobian matrix, H, which takes the derivative of the $u_{l}$ with respect to σ [14]. In other words, H is a mapping function that predicts the perturbation in $u_{l}$, caused by variations in σ. This relation is expressed as

(5) $$△u_{l}=H△\sigma \;$$

In practice, $△u_{l}$ is taken as the difference in electrode voltage data measured between a time interval ΔT, and the inversely calculated △σ is the relative variation in conductivity distribution between the period ΔT. This method is known as time difference imaging. Notably, $u_{n}$ is a part of the forward solution in (4), but it is not associated with H in the inverse problem; therefore, the system of equations described by (5) is underdetermined. A unique solution cannot be determined. To accommodate this problem, Tikhonov-styled regularization [16] is typically employed by minimizing the least square error described by the cost function described by

(6) $$e_{\mathrm{LS}}={\left|H△\sigma -△u_{l}\right|}^{2}+\lambda^{2}{\left|R△\sigma \right|}^{2}$$

where $e_{\mathrm{LS}}$ denotes the least square error, λ is the hyperparameter that controls the weight of the regularization, and R is a selected regularization matrix. Finally, a unique solution for which $e_{\mathrm{LS}}$ is minimized can be obtained as

(7) $$△\sigma ={\left(H^{T}H+\lambda^{2}R^{T}R\right)}^{-1}H^{T}△u_{l}$$

The summarized operating principle of EIT-based E-skins for touch sensing is illustrated in Figure 1c. Briefly, when a pressure or strain is applied to the sensing material, it induces a change in the conductivity distribution of the material. This change results in a difference in electric potential distribution and therefore causes a variation in the voltage data measured from the electrodes. This process is numerically modeled by the forward problem using FEM. Conversely, the inverse problem seeks a best-fitted conductivity solution by mapping the electrode voltage data backward using the Jacobian matrix and the method of regularization [17]. Finally, the conductivity solution reconstructed from the inverse problem can be used to interpret the presence of the applied mechanical stimuli in terms of planar spatial distribution.

### 2.2. Material Characterization and Sensor Fabrication

**Figure 2:**
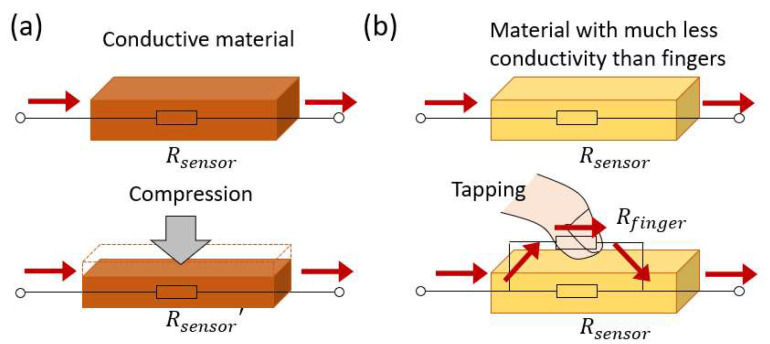
The mechanism for detecting a touch. (**a**) The conventional touch-detection mechanism used by sensors in previous works: physical compression causes material deformation, which leads to a change in the electrical resistance. (**b**) The proposed touch-detection mechanism: a finger contacting a less conductive material partially short-circuit the conductive path and significantly alters the measured signals.

As mentioned above, EIT-based touch sensors rely on the piezoresistive effect of pressure-sensitive materials, which converts the pressure exerted by the contacting objects into a change in the electrical conductivity of the sensor, as depicted in Figure 2a. Suppose the contact is so subtle that it does not exert a considerable force. In that case, the resulting change in the electrical conductivity of the sensor is insignificant, and the corresponding perturbation in the measured voltage data is tiny. As a result, it is difficult to detect or localize the contact based on the conductivity image reconstructed from the voltage data.

In order to effectively detect subtle contacts made by a small or negligible force, we took an approach that is distinguished from the piezoresistive-sensing mechanism commonly adopted by previous works. The proposed concept is illustrated in Figure 2b. The sensor can be fabricated using a material with considerably less electrical conductivity than human skin. When an electrical contact is made between a finger and the sensor, a more conductive path through the fingertip is formed to partially short-circuit the less conductive path through the sensing material. Therefore, when EIT is applied to such a material, the physical compression from a finger is not necessarily required to cause a conductivity perturbation at the contacting area. The conductivity perturbation is no longer sensitive to the physical compression; instead, it is now predominantly sensitive to the short-circuited conductive path along the contacting area between the finger and the sensor. Apart from human fingers, contacts made by other objects can also be detected, given that the contacting objects must be electrically conductive. Contacts made by non-conductive objects cannot be detected using the short-circuiting approach. Examples may include PVC gloves and skin with a thick layer of callus.

**Figure 3:**
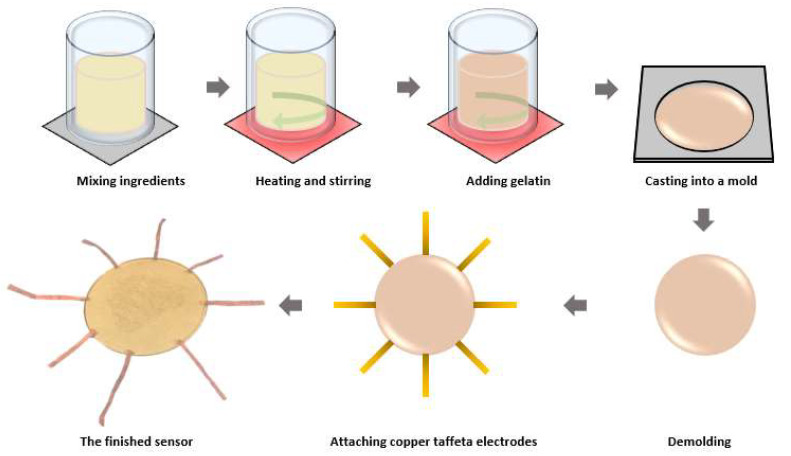
The fabrication procedure of the hydrogel-based touch sensor.

The equivalent electrical conductivity of the human skin, including its underlying layers, is approximately 0.090 S/m [18]. This value may vary among individuals and depends on multiple factors, such as skin region, water content, skin diseases, and temperature [19,20,21]. In order to detect subtle finger contact, we prepared a sensor using a hydrogel material with significantly less conductivity than this approximated conductivity value. The hydrogel was made of gelatine (Clear and Unflavoured Natural Gelatin, Davis Food Ingredients, Auckland, New Zealand), glycerol (HealthE glycerol BP liquid, Jaychem Industries Ltd., Auckland, New Zealand), and deionized water (RS PRO deionized water for PCB, RS Components Ltd., Corby, UK). Gelatine is a biopolymer extracted from animal collagen [22]; it can absorb water to form a hydrogel. Glycerol was used as a preservative to keep the hydrogel long-lasting without needing insulation from the air. The fabrication procedure is illustrated in Figure 3. The liquid ingredients, which consisted of 40 g of deionized water and 40 g of glycerol, were mixed in a beaker. The mixture was stirred using a glass rod and heated to 80 °C using a hot plate. Then, 20 g of gelatine powder was added to the mixture. The mixture was continuously stirred at 80 °C until the gelatine powders were completely dissolved. The obtained aqueous solution was cast into a disc-shaped mold with a diameter of 10 cm and cooled at room temperature (15 °C) for 3 h to allow gel formation. After the gelation process was finished, the hydrogel was released from the mold. The obtained hydrogel has a thickness of 2 mm and a diameter of 10 mm. Copper taffeta strips were directly attached to the hydrogel by pressing the strips against the hydrogel using a soldering iron briefly. The heat allowed slight melting of the hydrogel so that the adhesion was realized by the self-healing property [23] of the physically crosslinked hydrogel.

The electrical conductivity of the hydrogel was verified with an MFIA Impedance Analyzer (Zurich Instruments AG, Zurich, Switzerland) under a sweep frequency from 1 kHz to 1 MHz. The electrical conductivity of the hydrogel was measured to be between 0.0178 and 0.0201 S/m. It was observed that the hydrogel could maintain its shape and dimension without noticeable deformation under room temperature (i.e., 15 °C) for at least 14 days.

### 2.3. Data Acquisition

**Figure 4:**
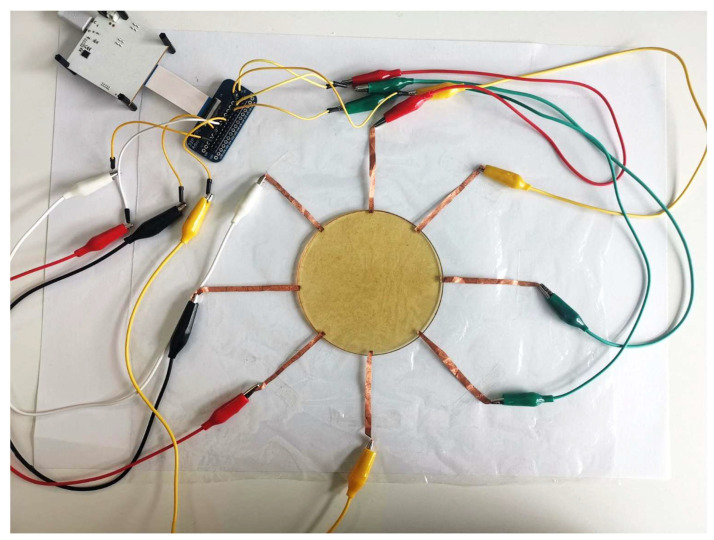
The sensor is connected to a data acquisition device to allow data collection.

In order to implement tomographic imaging on the acquired hydrogel sensor, we used Spectra (Mindseye Biomedical, San Francisco, CA, USA) as the data acquisition system to perform current excitation and voltage measurements. The system is based on an ADUCM350 microcontroller for impedance analysis with a 16-bit resolution and a 160 kSPS sampling rate. The hydrogel sensor was connected to Spectra, as shown in Figure 4.

**Figure 5:**
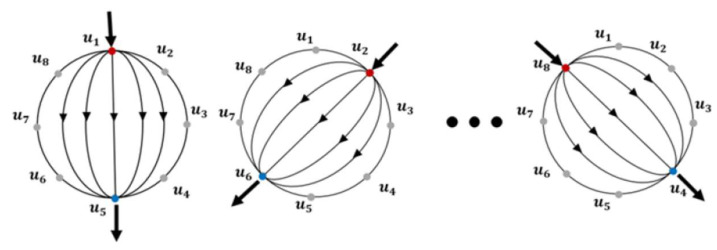
The opposite current excitation strategy: an electrical current flows through the sensitive region via two electrodes at the opposite ends sequentially.

We adopted the opposite current excitation strategy [24] to drive the sequence of voltage measurement. The opposite strategy is illustrated in Figure 5. The opposite pattern allows the current to flow across the domain and passes the central region more uniformly than the other commonly used adjacent strategy; therefore, touches applied to the interior region of the sensor can be detected more efficiently. Following this strategy, an alternating excitation current of 25 kHz was passed through two electrodes located at opposite ends of the domain; meanwhile, voltages at the remaining electrodes were measured. Once this step was completed, the excitation channel was switched to the next pair of electrodes by controlling the embedded multiplexers. This process was repeated for all opposite pairs of electrodes so that the voltage data at each electrode during each current injection was recorded.

The recorded voltage data was processed by a computer. We used MATLAB (MathWorks Inc., Natick, MA, USA) to perform data processing and execute the reconstruction algorithms described in Section 2.1. The total variation regularization was selected to solve the inverse problem, as this method does not penalize the image discontinuities [25] that are typically present in touch sensing. The reconstruction converts the recorded voltage data to estimated conductivity distribution, from which touch can be detected and localized.

## 3. Experiments

A series of experiments were designed to demonstrate the performance of the proposed touch sensor. First, the sensor was tested to detect finger contacts, including single and double touches. Second, coins of different sizes were placed onto the sensor to exhibit its capability of distinguishing the difference in the contact area. Finally, conductive fabrics with negligible weight were placed onto the sensor to demonstrate its capability of detecting subtle contact made by conductive objects without exerting a force.

### 3.1. Detecting Single and Double Touches

**Figure 6:**
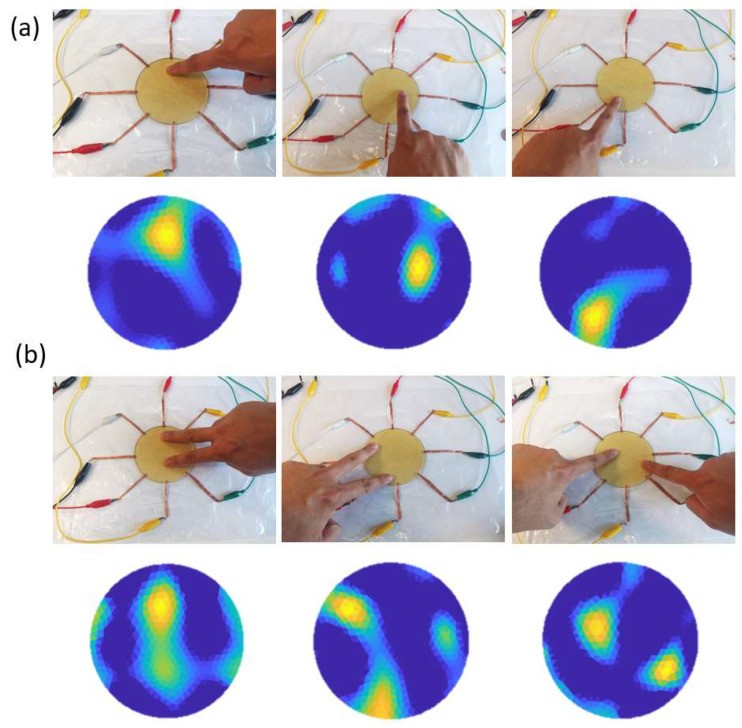
The proposed sensor is used for detecting finger contacts. (**a**) Conductivity images showing contact made by a single finger. (**b**) Conductivity images showing contact made by two fingers simultaneously.

Contacts were made by directly tapping the sensor with single or double fingers at various locations on the sensor surface. The contacts were recorded using a camera and compared with the conductivity images reconstructed using EIT. Since contacts made by fingers or other conductive objects only increase the local conductivity of the sensor, conductivity variations that were estimated to have negative values were set to zero to make the contacts more visible. The results are demonstrated in Figure 6. In Figure 6a, contacts were made at the top, middle, and bottom parts of the sensor by a single finger, respectively. The reconstructed conductivity image showed a strong correlation with the contact points. In Figure 6b, contacts were made using two fingers simultaneously at different locations. The images produced by EIT successfully captured the changes in the electrical conductivity at the corresponding positions. These results confirmed that the proposed sensor was capable of detecting single and double contacts. It is worth noting that, due to the intrinsic low-resolution limitation of EIT, multiple contacts applied to the sensitive region should be separated by a distance of at least 3 cm to allow their successful detection.

### 3.2. Distinguishing Contact Area

**Figure 7:**
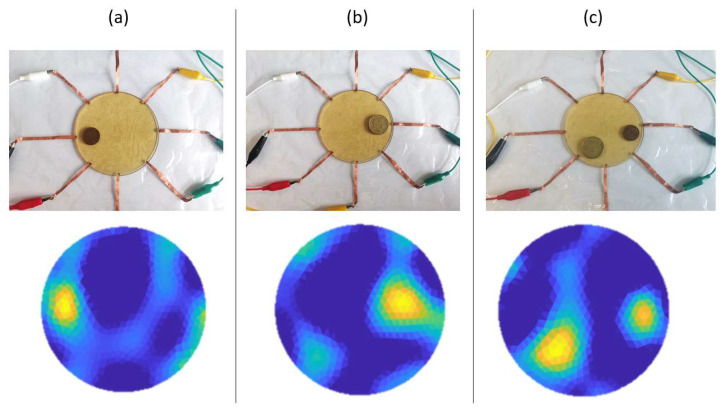
The proposed sensor can distinguish the difference in the contact area. (**a**) A 10 cents coin with a diameter of 20.5 mm is placed onto the sensor. (**b**) A 2 dollars coin with a diameter of 26.5 mm is placed onto the sensor. (**c**) Both coins are placed onto the sensor.

The sensor’s ability to distinguish the difference in contact area was examined. Coins of different dimensions, including a ten-cent coin with a diameter of 20.5 mm and a two-dollar coin with a diameter of 26.5 mm, were placed on top of the sensor to produce electrical contact with different areas. The two coins have weights of 3.3 g and 10 g, respectively. Conductivity images were reconstructed using EIT and are demonstrated in Figure 7. In Figure 7a,b, conductivity images corresponding to the contacts made by the two coins separately. It can be seen that, although the silhouettes of the obtained conductivity contrasts were not precisely matched with the actual contact area, the difference between the sizes of the two silhouettes was roughly exhibited, indicating the sensor could identify the relatively larger or smaller contacts when multiple contacts are made. This ability was more clearly demonstrated in Figure 7c, where the two coins were placed onto the sensor at the same time. The obtained conductivity image demonstrates a clear difference between the sizes of the two conductivity contrasts, confirming that the proposed sensor could roughly distinguish different sizes of contact area.

### 3.3. Detecting Subtle Contact

**Figure 8:**
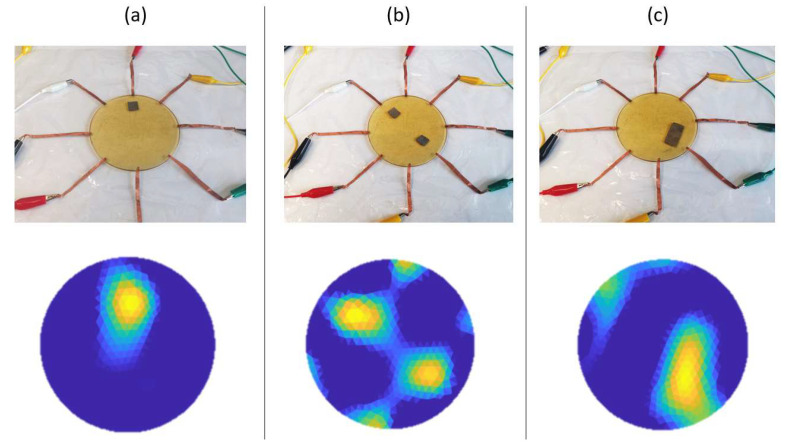
The proposed sensor is used to detect subtle contacts made by silver fiber fabric strips with negligible weight. (**a**) A single strip with a 1 ${\mathrm{cm}}^{2}$ area is placed onto the sensor. (**b**) Two strips with the same 1 ${\mathrm{cm}}^{2}$ area are placed onto the sensor. (**c**) A strip with a 3 ${\mathrm{cm}}^{2}$ area is placed onto the sensor.

The sensor was tested for its ability to detect subtle contacts. Silver fiber fabric strips that possess negligible weight were placed on top of the sensor to mimic a forceless finger touch. Square-shaped strips of two dimensions were used: the smaller strip has an area of approximately 1 ${\mathrm{cm}}^{2}$ and weighed about 11 mg; the larger strip has an area of approximately 3 ${\mathrm{cm}}^{2}$ and weighed about 35 mg. The strip placement and the corresponding conductivity images produced by EIT are demonstrated in Figure 8. In Figure 8a, the smaller strip was placed on top of the sensor to mimic a single forceless touch, which could be detected from the obtained conductivity image. In Figure 8b, two strips of the same 1 ${\mathrm{cm}}^{2}$ area were placed onto the sensor to mimic double forceless contacts applied simultaneously. The obtained conductivity image successfully exhibited the presence of both contacts. In Figure 8c, the larger strip was placed in contact with the sensor. The corresponding conductivity image exhibited an evidently larger contact area than the results in Figure 8b,c. These results confirmed that the proposed sensor could effectively detect contacts without requiring a noticeable force. It was worth noting that forming an electrical path between the sensor and the contacting objects does not necessarily require applying a force; therefore, the proposed method could theoretically achieve touch detection even if there is no force exerted on the sensor.

## 4. Conclusions

This paper proposed an EIT-based electronic-skin sensor for enabling touch detection. The sensor is suitable for localizing contacts made by human fingers or other electrically conductive objects anywhere within its sensitive area. The sensor supports the detection of single and multiple contacts and can roughly identify the difference in the contact area. This work features a novel touch detection mechanism enabled via a short-circuiting approach, which is distinct from the piezoresistive approach typically adopted by previous works. It provides a new perspective for achieving EIT-based touch detection, which reveals that the EIT-based touch sensing technique can utilize the electrical contact between the sensing material and the conductive object. Compared to the conventional piezoresistive sensing approach, the newly proposed method possesses the advantage of enabling touch detection without requiring any noticeable force. To the knowledge of the authors, the proposed approach has not been previously reported. It may be particularly beneficial for the scenario where detecting subtle contacts without causing mechanical deformation of the target object is preferred.

## Data Availability

Not applicable.
